# Lung function and risk of incident dementia: A prospective cohort study of 431,834 individuals

**DOI:** 10.1002/alz.088820

**Published:** 2025-01-09

**Authors:** Liu Yang, Jin‐Tai Yu

**Affiliations:** ^1^ Fudan University, Shanghai, Shanghai China; ^2^ National Center for Neurological Disorders, Shanghai China; ^3^ Huashan Hospital, Fudan University, Shanghai China

## Abstract

**Background:**

Whether lung function prospectively affects cognitive brain health independent of their overlapping factors remains largely unknown. This study aimed to investigate the longitudinal association between decreased lung function and cognitive brain health and to explore underlying biological and brain structural mechanisms.

**Method:**

This population‐based cohort included 43,1834 non‐demented participants with spirometry from the UK Biobank. Cox proportional hazard models were fitted to estimate the risk of incident dementia for individuals with low lung function. Mediation models were regressed to explore the underlying mechanisms driven by inflammatory markers, oxygen‐carrying indices, metabolites, and brain structures.

**Result:**

During a follow‐up of 3,736,181 person‐years (mean follow‐up 8.65 years), 5,622 participants (1.30%) developed all‐cause dementia, which consisted of 2,511 Alzheimer’s dementia (AD) and 1,308 Vascular Dementia (VD) cases. Per unit decrease in lung function measure was each associated with increased risk for all‐cause dementia (forced expiratory volume in 1 s [liter]: hazard ratio [HR, 95%CI], 1.24 [1.14‐1.34], P = 1.10 × 10^−07^; forced vital capacity [liter]: 1.16 [1.08‐1.24], P = 2.04 × 10^−05^; peak expiratory flow [liter/min]: 1.0013 [1.0010‐1.0017], P = 2.73 × 10^−13^). Low lung function generated similar hazard estimates for AD and VD risks. As underlying biological mechanisms, systematic inflammatory markers, oxygen‐carrying indices, and specific metabolites mediated the effects of lung function on dementia risks. Besides, brain grey and white matter patterns mostly affected in dementia were substantially changed with lung function.

**Conclusion:**

Life‐course risk for incident dementia was modulated by individual lung function. Maintaining optimal lung function is useful for healthy aging and dementia prevention.

